# Trade‐offs between reducing complex terminology and producing accurate interpretations from environmental DNA: Comment on “Environmental DNA: What's behind the term?” by Pawlowski et al., (2020)

**DOI:** 10.1111/mec.15942

**Published:** 2021-05-25

**Authors:** Naiara Rodriguez‐Ezpeleta, Olivier Morissette, Colin W. Bean, Shivakumara Manu, Pritam Banerjee, Anaïs Lacoursière‐Roussel, Kingsly C. Beng, S. Elizabeth Alter, Fabian Roger, Luke E. Holman, Kathryn A. Stewart, Michael T. Monaghan, Quentin Mauvisseau, Luca Mirimin, Owen S. Wangensteen, Caterina M. Antognazza, Sarah J. Helyar, Hugo de Boer, Marie‐Eve Monchamp, Reindert Nijland, Cathryn L. Abbott, Hideyuki Doi, Matthew A. Barnes, Matthieu Leray, Pascal I. Hablützel, Kristy Deiner

**Affiliations:** ^1^ AZTI Basque Research and Technology Alliance (BRTA), Marine Research Sukarrieta Spain; ^2^ Direction de l'expertise sur la Faune Aquatique, Ministère des Forêt de la Faune et des Parcs Québec QC Canada; ^3^ Scottish Centre for Ecology and the Natural Environment Institute of Biodiversity, Animal Health and Comparative Medicine University of Glasgow Glasgow UK; ^4^ Laboratory for the Conservation of Endangered Species CSIR‐Centre for Cellular and Molecular Biology Hyderabad India; ^5^ Department of Biomedical Sciences National Chung Cheng University Chiayi Taiwan; ^6^ Department of Earth and Environmental Sciences National Chung Cheng University Chiayi Taiwan; ^7^ Government of Canada Department of Fisheries and Oceans St. Andrews Biological Station St. Andrews NB Canada; ^8^ Department of Ecosystem Research Leibniz Institute of Freshwater Ecology and Inland Fisheries (IGB) Berlin Germany; ^9^ Department of Biology and Chemistry California State University Monterey Bay Seaside CA USA; ^10^ Centre for Environmental and Climate Research (CEC) Lund University Lund Sweden; ^11^ School of Ocean and Earth Science National Oceanography Centre Southampton University of Southampton Southampton UK; ^12^ Institute for Biodiversity and Ecosystem Dynamics University of Amsterdam the Netherlands; ^13^ Institut für Biologie Freie Universität Berlin Berlin Germany; ^14^ Natural History Museum University of Oslo Oslo Norway; ^15^ Department of Natural Sciences School of Science and Computing Galway‐Mayo Institute of Technology Galway Ireland; ^16^ Norwegian College of Fishery Science UiT the Arctic University of Norway Tromsø Norway; ^17^ Department of Theoretical and Applied Sciences University of Insubria Varese Italy; ^18^ Institute of Global Food Security (IGFS) School of Biological Sciences Queen's University Belfast Belfast UK; ^19^ Natural History Museum University of Oslo Oslo Norway; ^20^ Department of Biology McGill University Montreal QC Canada; ^21^ Marine Animal Ecology Group Wageningen University Wageningen The Netherlands; ^22^ Pacific Biological Station, Fisheries and Oceans Canada Nanaimo BC Canada; ^23^ Graduate School of Simulation Studies University of Hyogo Kobe Japan; ^24^ Department of Natural Resources Management Texas Tech University Lubbock TX USA; ^25^ Smithsonian Tropical Research Institute Smithsonian Institution Panama City Panama; ^26^ Flanders Marine Institute (VLIZ) Ostend Belgium; ^27^ Department of Environmental Systems Science ETH Zurich Zurich Switzerland

**Keywords:** clear terminology, ecology of eDNA, extra‐organismal DNA, organismal DNA

## Abstract

In a recent paper, “Environmental DNA: What's behind the term? Clarifying the terminology and recommendations for its future use in biomonitoring,” Pawlowski *et al*. argue that the term eDNA should be used to refer to the pool of DNA isolated from environmental samples, as opposed to only extra‐organismal DNA from macro‐organisms. We agree with this view. However, we are concerned that their proposed two‐level terminology specifying sampling environment and targeted taxa is overly simplistic and might hinder rather than improve clear communication about environmental DNA and its use in biomonitoring. This terminology is based on categories that are often difficult to assign and uninformative, and it overlooks a fundamental distinction within eDNA: the type of DNA (organismal or extra‐organismal) from which ecological interpretations are derived.

## 1 EDNA SHOULD BE USED TO REFER TO THE TOTAL POOL OF DNA ISOLATED FROM THE ENVIRONMENT

Clear and unambiguous scientific terminology is important to communicate science, particularly when misunderstanding or miscommunications can lead to costly ramifications (Gouran et al., 1986; Jerde, 2019; Mahon et al., 2013). Hence, we applaud Pawlowski et al. (2020) for highlighting inconsistencies in the use of the term “environmental DNA” (eDNA) and their implications for biomonitoring. As described by the authors, these inconsistencies stem from some researchers using the term to refer to any DNA collected from an environmental sample without first isolating targeted organisms (e.g., Stat et al. (2017)), while others use it to refer only to extra‐organismal DNA released by macro‐organisms into the environment (e.g., Fraija‐Fernández et al. (2020)). Although some of us have previously advocated for eDNA to be defined as extra‐organismal DNA, the value of which is effectively refuted by Pawlowski et al. (2020), we agree with Pawlowski et al. (2020) that environmental DNA should be defined in the broadest sense.

However, the recommendation to employ a standard two‐level terminology in eDNA studies, first indicating the environmental origin of the DNA collected (e.g., water, sediment, biofilm, soil) and second indicating the taxa (e.g., fish, diatom, bacteria) targeted by polymerase chain reaction (PCR), does not align with the overall purpose of improving clarity in eDNA biomonitoring. The reason is that it does not account for the distinction between the different types of eDNA (organismal and extra‐organismal), which is the level of classification that can have a strong impact on eDNA data interpretation. While Pawlowski et al. (2020) discount this, we argue there is a need to be clear about the type of eDNA that is being evaluated in any given study and this is the reason for why the term has been described in the broad and narrow sense.

## 2 EDNA IS COMPOSED OF ORGANISMAL AND EXTRA‐ORGANISMAL DNA

**FIGURE 1:**
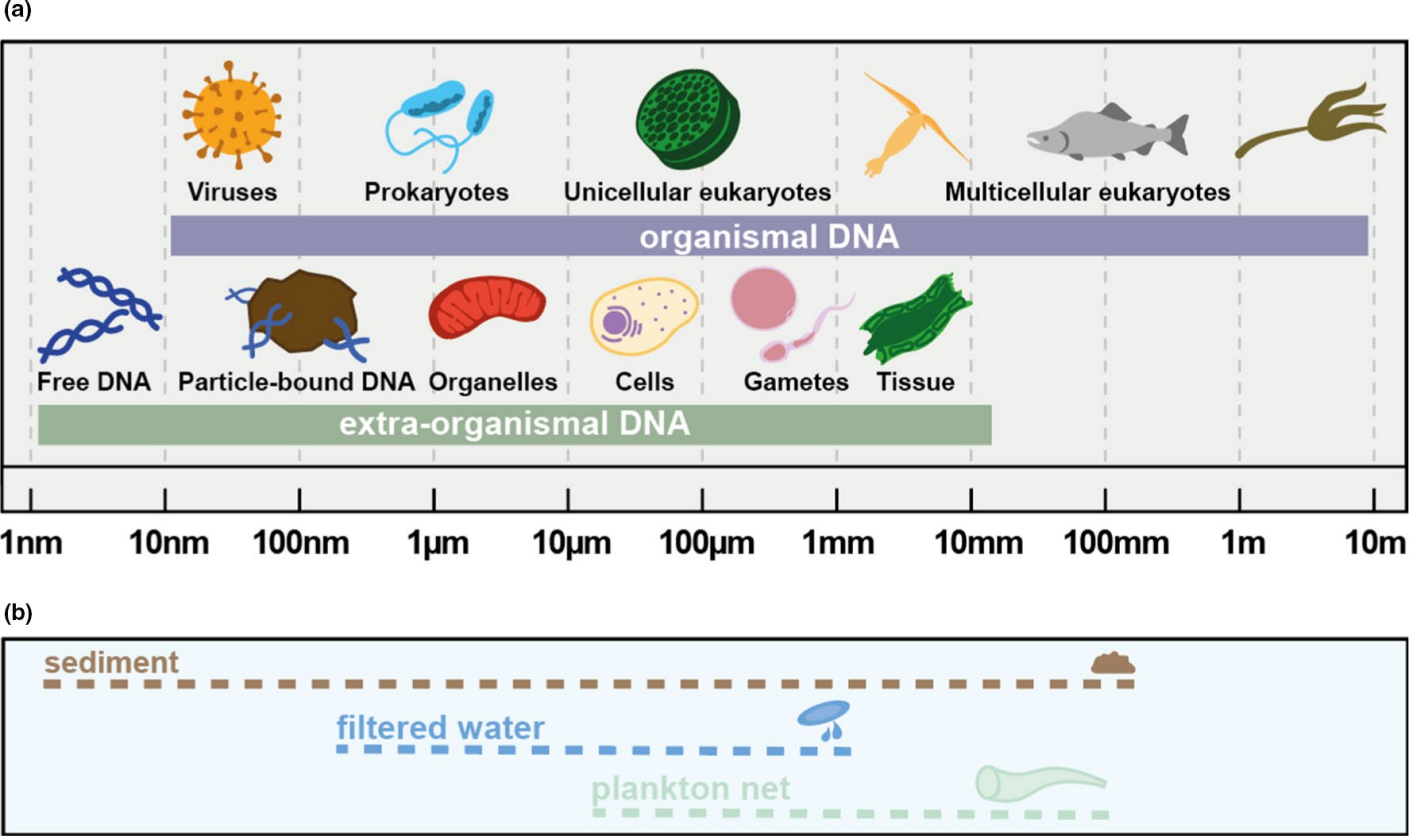
(a) Types of environmental DNA (organismal and extra‐organismal, including extracellular) with possible sources and approximate size ranges. (b) Illustrative examples of sampling methods with intended captured particle size ranges

Environmental DNA can be classified into two types (Figure 1a): organismal DNA and extra‐organismal DNA, the latter also including extracellular DNA (Barnes & Turner, 2016; Bohmann et al., 2014; Taberlet et al., 2012; Torti et al., 2015). Organismal DNA is sourced from whole individuals most probably alive at the time of sampling; as such, this type of eDNA is typically of high quality and significant quantity. In contrast, extra‐organismal DNA originates from a variety of sources and thus is of highly variable quality and quantity. For example, extra‐organismal DNA can come: (i) from biological material shed from an organism as part of tissue replacement or metabolic waste (Allan et al., 2020); (ii) as biologically active propagules such as gametes, pollen, seeds or spores (Stewart, 2019); or (iii) as a result of cell lysis or cell extrusion (Pietramellara et al., 2009). The latter processes results in extracellular DNA, which can persist in the environment on its own or be adsorbed onto surface‐reactive particles such as humic substances, clay, silt or sand (Levy‐Booth et al., 2007; Pietramellara et al., 2009). Environmental DNA samples are therefore composed of a complex mixture of both types of DNA (i.e., organismal and extra‐organismal) from various sources and in varying proportions (Taberlet et al., 2012).

## 3 EDNA CAN BE ENRICHED FOR DIFFERENT SOURCES AND TYPES

Generally, not all DNA present in a studied environment is required to address a given research question or is used for an application, and successive steps of enrichment for specific types or sources of eDNA are usually applied. For example, eDNA from a large variety of taxonomic groups can be found as organismal or extra‐organismal DNA (types) in the environment (Figure 1a) and can be obtained in many ways from aquatic, aerial and terrestrial environments (Deiner et al., 2017). The first step is performed at the sampling level, where typically the collected material is passed through filters, meshes or nets to retain organisms, organismal debris or particles of a desired size (Figure 1b). Notably, this step does not imply a separation of DNA types or taxonomic groups because different sources and types of DNA overlap in size (Figure 1a) and because of the “sticky” nature of eDNA to bind other particles (Barnes et al., 2020). A subsequent enrichment can be performed during laboratory work through PCR or sequence capture using taxon‐specific primers or probes (Jensen et al., 2020). However, this step is not perfect; a fraction of nontarget taxa DNA can also be amplified, and target taxa DNA can be missed. Finally, DNA sequences from particular taxa can be selected at the analysis/interpretation step by considering only those sequences belonging to a given taxonomic group.

The particular methods applied at each of these enrichment steps will determine the final data set used for ecological inferences, but these methods evolve and are not in themselves completely deterministic. For example, “water eDNA amplified for metazoans” could refer either to organismal DNA collected through a plankton net containing fish larvae and zooplankton, or to extra‐organismal DNA collected through a 0.45‐μm pore size filter containing tissue, scales or cellular debris from fish and zooplankton.

## 4 ECOLOGICAL INTERPRETATIONS SHOULD CONSIDER DNA TYPE

While it is currently impractical to separate and independently analyse organismal and extra‐organismal DNA, the distinction between the two types is nonetheless crucial for ecological hypothesis‐testing and data interpretation. Organismal DNA is often targeted when a living community of organisms is studied, asking questions about specific habitat, the functional role of communities or community assembly processes driven by abiotic factors and biotic interactions. Here, the chances of misleading data (i.e., the species was not in that environment at that time and place) are likely to be minimal. Instead, work focusing on extra‐organismal DNA is more prone to misinterpretations about organismal distribution due to potential long‐distance transport from source populations (Lacoursière‐Roussel & Deiner, 2021). The processes regulating the presence of extra‐organismal DNA in the environment and its detection in the laboratory are more stochastic. As a result, studies targeting this type of eDNA require a sampling design with in‐depth replication and extra attention to potential sources of contamination, and need to be cognizant that the results are less likely to be definitive about species presence or absence at the time of sampling.

In eDNA studies, extra‐organismal DNA is increasingly targeted for the indirect detection of (often macro‐) organisms without destroying their natural habitats or harming individuals: for example, detecting fish taxa from eDNA extracted from water (Antognazza et al., 2019; Fraija‐Fernández et al., 2020). Here, any link between the presence of a species' DNA and the presence of a living individual or population in the local area is implied. While a recent meta‐analysis found that fish diversity estimated using eDNA agrees closely with estimates using conventional methods of capturing or observing the fish (McElroy et al., 2020), absolute conclusions about space and time inferences made from extra‐organismal DNA are not yet possible. To make such a link accurate, an understanding of the “ecology” of extra‐organismal DNA (Barnes & Turner, 2016) is crucial, which requires knowledge of the often site‐specific processes governing its production, transportation and degradation rate in the environment.

While separating the different eDNA types in practice remains a challenge, researchers using eDNA need to be clear about their intent. Specifically, we need to clearly report the methodological choices made to target one type of eDNA or another (whether by sampling, laboratory treatment or bioinformatics), make informed speculations about the likelihood of succeeding with that target, and acknowledge the limitations of the data we generate. If we target extra‐organismal DNA, we also need to consider what process(es) we hypothesize govern the transport between the temporal and spatial bounds of detected DNA and what inferences we can therefore make from its detection.

## 5 CONCLUSIONS

In summary, we agree with Pawlowski et al. (2020) that eDNA should be defined in the broadest sense, but do not agree that the formal adoption of their additional proposed nomenclature will improve clarity in communication or reduce confusion around the use of the term eDNA. We suggest instead that scientists carefully and clearly identify the type of DNA being targeted for analysis (Figure 1) based on the existing terminology of organismal and extra‐organismal DNA. This explicit stated intention would then clearly inform study design, sampling strategies, analytical choices and data interpretation to avoid potential biases and promote valid inferences. Because none of these choices and strategies are perfect in their detection of a particular type of DNA and in the place of a field‐specific nomenclature, we suggest that in the methods sections of studies, authors should clearly describe the sampling strategy including the targeted size classes and taxa and whether taxa were targeted in any way during sampling, laboratory analysis (PCR, capture), data analysis (sequence selection) or some combination thereof. We feel that improvement of the field is a shared responsibility among researchers, reviewers, editors and managers and support the development and application of best practices in the acquisition and reporting of eDNA data (Goldberg et al., 2016) as the best way to improve clarity.

## AUTHOR CONTRIBUTIONS

N.R.E. and K.D. conceived the idea. All authors contributed to the discussion and wrote the manuscript. L.E.H. constructed the figure with input from all authors.

## Data Availability

Not applicable.
